# Low-Intensity Virtual Reality Exercise for Caregivers of People with Mild Cognitive Impairment: A Pilot Study

**DOI:** 10.3390/jfmk10030353

**Published:** 2025-09-16

**Authors:** Maria Grazia Maggio, Raffaela Maione, Silvia Migale, Antonino Lombardo Facciale, Luca Pergolizzi, Piero Buonasera, Bartolo Fonti, Mirjam Bonanno, Giulia Pistorino, Paolo De Pasquale, Rocco Salvatore Calabrò

**Affiliations:** 1 IRCCS Centro Neurolesi Bonino-Pulejo, S.S. 113 Via Palermo, C. da Casazza, 98124 Messina, Italy; mariagrazia.maggio@irccsme.it (M.G.M.); raffaela.maione@irccsme.it (R.M.); silvia.migale@irccsme.it (S.M.); antonino.lombardo@irccsme.it (A.L.F.); luca.pergolizzi@irccsme.it (L.P.); piero.buonasera@irccsme.it (P.B.); bartolo.fonti@irccsme.it (B.F.); mirjam.bonanno@irccsme.it (M.B.); roccos.calabro@irccsme.it (R.S.C.); 2 Department of Clinical and Experimental Medicine, University of Messina, p.zza Pugliatti, 98122 Messina, Italy; giulia.pistorino@studenti.unime.it

**Keywords:** informal caregivers, caregiver burden, virtual reality (VR), physical training, psychological well-being, telerehabilitation, neurorehabilitation

## Abstract

**Background:** Informal caregivers of individuals with mild cognitive impairment (MCI) experience high levels of psychological and physical stress, with limited access to supportive interventions and time constraints. Virtual Reality (VR) technologies may provide brief and accessible opportunities to support caregiver well-being, particularly during waiting periods in clinical settings. This pilot study aimed to explore the potential of a semi-immersive VR intervention to enhance psychological well-being in informal caregivers. **Methods:** This non-randomized pilot study investigated the effects of a semi-immersive VR-based physical training program (K-HERO^®^) on psychological well-being and coping strategies in informal caregivers. Participants were recruited from January to May 2025 at the IRCCS Centro Neurolesi “Bonino-Pulejo” (Messina, Italy), and the intervention was delivered individually in a dedicated room within the rehabilitation facility, while caregivers accompanied their relatives to treatment sessions. Ten caregivers completed six sessions (30–40 min each). The study was conducted in accordance with TREND reporting guidelines. Pre- and post-intervention assessments included the State-Trait Anxiety Inventory (STAI), Perceived Stress Scale (PSS), COPE Inventory, Caregiver Burden Inventory (CBI), and International Physical Activity Questionnaire (IPAQ). Visual Analog Scales and instrumental data from the VR system were used to monitor physical performance and user experience. Non-parametric statistics were applied. **Results:** Significant reductions were observed in avoidance (*p* = 0.033) and social support-based (*p* = 0.023) coping strategies. Differences emerged based on caregiver-patient relationships: parental caregivers showed increased anxiety, while offspring caregivers showed improvements. The intervention was well tolerated, with high usability and no adverse events reported. **Conclusions:** A short, structured, VR-based intervention delivered during clinical waiting periods may effectively reduce maladaptive coping strategies and support emotional well-being in informal caregivers. These findings highlight the potential of brief digital interventions in real-world care contexts. Larger randomized studies are needed to validate these preliminary findings and personalize interventions to different caregiver profiles.

## 1. Introduction

Informal caregivers are often family members or close friends. They provide essential unpaid support to individuals living with chronic illnesses or disabilities [1,2]. Their role typically includes assistance with daily tasks and medical needs. However, caregivers are also responsible for emotional, organizational, and logistical support, which can significantly impact caregivers’ physical and psychological health [3,4]. Although crucial to both care recipients and the sustainability of healthcare systems, informal caregiving frequently comes at a high personal cost. A growing body of literature highlights the health risks associated with prolonged caregiving. Informal caregivers are more likely to experience psychological distress [5,6], and are vulnerable to physical health problems, such as impaired immune responses and cardiovascular issues [7,8,9]. Their responsibilities are often multifaceted and challenging, encompassing tasks such as mobility support, medication management, coordination with healthcare providers, and financial oversight [10]. In cases of neurodegenerative conditions, caregiving typically intensifies over time, involving progressively complex and physically intensive activities, including feeding, bathing, toileting, and lifting [11].

The impact of caregiving is often conceptualized in terms of two interconnected constructs: caregiver burden and caregiver strain [2]. Caregiver burden refers to the cumulative toll on physical, emotional, and financial well-being, often associated with increased workload, changing family dynamics, and economic hardship, leading to physical exhaustion and emotional fatigue [12]. In contrast, caregiver strain reflects the multidimensional effects of caregiving, physical, psychological, emotional, and social [13].

Given the high levels of psychological and physical strain experienced by informal caregivers, there is a compelling need for accessible and effective interventions aimed at supporting their well-being. To help reduce stress and promote resilience, caregivers benefit from simple, brief, and accessible opportunities for self-care, especially during the overlooked waiting periods while their loved ones receive treatment. These unstructured moments represent valuable time windows to implement low-demand, restorative activities that can enhance caregivers’ emotional balance, perceived control, and sense of inclusion within the care process [14,15,16]. Literature on caregiver support emphasizes the importance of integrating micro-interventions into daily routines to reduce the psychological load and prevent long-term burnout [17,18].

In this context, novel technologies such as virtual reality (VR) may offer immediate, scalable interventions [19]. VR and augmented reality (AR) are rapidly developing technologies that are increasingly being used in the medical field [20]. VR immerses the user completely in a virtual 3D environment, while AR enhances the real world by overlaying virtual elements to provide additional information [20]. Both require the use of special VR/AR headsets [21]. In medicine, these technologies are already being applied in well-researched areas such as cardiovascular care [22] and neurosurgery [23]. Their use is also expanding in intensive care medicine, with potential benefits for both healthcare professionals and patients [20]. For medical staff, VR offers a safe space to learn and practice complex procedures in intensive care [24], while AR supports decision-making, integrating helpful information before and during interventions [25]. From the patient’s perspective, VR can help reduce stress during intensive care stays, offering distraction from pain for both adults and children [26,27]. Moreover, VR-based gamified activities have demonstrated potential in supporting cognitive and motor function enhancement [28]. Overall, VR and AR can be used by different users at various stages of the medical process across different applications [21].

One of these systems is K-HERO^®^ (Drop Digital Health S.r.l., Rome, Italy), an innovative medical technology designed to overcome common challenges in telerehabilitation and enable independent physical training while ensuring clinical accuracy. The system integrates artificial intelligence to monitor functional and clinical parameters, including mobility, balance, posture, joint angles, and therapy adherence, allowing for continuous and personalized supervision.

This pilot study investigates the feasibility and potential of a semi-immersive VR intervention to promote psychological well-being among informal caregivers.

## 2. Materials and Methods

### 2.1. Study Design

This pilot study was conducted between January and May 2025 at the Day Hospital of the IRCCS Centro Neurolesi “Bonino-Pulejo” in Messina, Italy, in accordance with the TREND (Transparent Reporting of Evaluations with Nonrandomized Designs) guidelines [29] (see Supplementary Material S1). Participants were informal caregivers of patients diagnosed with mild cognitive impairment (MCI) undergoing rehabilitation at our center. Caregivers were recruited consecutively, aligned with the treatment period of their relatives.

Out of thirty caregivers approached, twelve consented to participate. Two participants were subsequently excluded (one due to the care recipient discontinuing physiotherapy, and one for methodological reasons related to sample balance), resulting in a final sample of ten caregivers who completed the full six-session intervention. The participant flow is detailed in Supplementary Figure S1.

The primary caregiver was defined as the individual assuming the main responsibility for the patient’s care, either at home or in other settings. Inclusion criteria for caregivers were age ≥18 years, absence of major medical or psychiatric disorders, and no evidence of severe cognitive impairment.

Although no randomization was applied, the study employed a clear pre-post evaluation design to assess within-subject changes over time. The methodology adhered to the core principles of the TREND guidelines, ensuring comprehensive reporting of setting, participants characteristics, intervention content, and outcome assessment.

The study was conducted in accordance with the Declaration of Helsinki. All participants provided voluntary, written informed consent prior to enrollment The study is part of a broader multi-year research project registered on ClinicalTrials.gov (ID: NCT07066137), coordinated by the IRCCS Centro Neurolesi “Bonino-Pulejo”, and was approved by the local Ethics Committee (Protocol code: IRCCSME 47/2023).

### 2.2. Outcome Measures

Outcomes were measured at two time-points: baseline (T0) and post-intervention (T1), using validated self-report instruments assessing psychological well-being, perceived stress, and coping strategies. Standardized psychometric assessments were administered using the Italian-validated versions of each tool. A licensed psychologist, blinded to the study’s aims, administered the questionnaires in a dedicated room that ensured privacy and minimized distractions. Data were collected according to a pre-defined protocol to minimize measurement bias.

An initial evaluation was conducted by a multidisciplinary team comprising a psychologist, physiotherapist, neurologist, and movement scientist, who collected demographic and eligibility data. Only caregiver data were analyzed in the study; no clinical or neuropsychological data from care recipients were included in the statistical analyses.

Anxiety symptoms were assessed using the State-Trait Anxiety Inventory (STAI), while coping strategies were evaluated with the COPE Inventory, which includes subscales for social support, avoidance, positive attitude, problem-oriented strategies, and transcendent orientation. Perceived stress was measured with the Perceived Stress Scale (PSS), and depressive symptoms with the Beck Depression Inventory-II (BDI-II). Caregiving-related burden was assessed using the Caregiver Burden Inventory (CBI). Physical activity levels were quantified with the International Physical Activity Questionnaire (IPAQ), which captures information on vigorous, moderate, and walking activities, along with a total physical activity score (see Table 1).

During each intervention session, the K-HERO^®^ system collected both subjective and objective data. At the end of each session, caregivers reported perceived execution difficulty, muscular effort, balance difficulty, and pain using visual analog scales (VAS) displayed on the device interface. Simultaneously, the system automatically recorded movement metrics, including left-side mobility, right-side mobility, and overall mobility, enabling quantitative monitoring of physical performance over time. Adherence and acceptability were monitored using session attendance logs and post-session feedback forms.

Physical activity was assessed using the International Physical Activity Questionnaire-Short Form (IPAQ-SF), with results expressed in Metabolic Equivalent of Task minutes per week (MET-min/week) and categorized as low (<600 MET-min/week), moderate (600–3000 MET-min/week), or high (>3000 MET-min/week) at both T0 and T1. Anxiety was evaluated with the State (Y1) and Trait (Y2) subscales of the State-Trait Anxiety Inventory (STAI), while coping strategies were assessed using the validated Italian version of the COPE Inventory.

All assessments were administered by a licensed psychologist, trained in standardized administration procedures and blinded to the study hypotheses. Adherence to the VR intervention was monitored by recording the number of completed sessions (out of six planned per participant), the duration of each session and the reasons for any missed sessions. Adverse events were systematically monitored through both self-reports and direct observation during sessions, and none were reported.

### 2.3. Procedures

The study involved twelve informal caregivers, of whom two were classified as dropouts. One caregiver attended three sessions, while the other completed only the first session because the care recipient discontinued physiotherapy. Caregivers were consecutively recruited between January and May 2025 while accompanying their relatives to rehabilitation at the IRCCS Centro Neurolesi “Bonino-Pulejo” (Messina, Italy).

Of the twelve caregivers initially enrolled, two participants did not complete the intervention: one discontinued after three sessions due to personal reasons, and the other withdrew after the first session due to the interruption of the patient’s physio-therapy treatment. Consequently, the final study sample consisted of ten caregivers who completed all six intervention sessions.

Demographic data indicated a heterogeneous sample, with a mean age of 58.9 years (standard deviation, SD = 18.2; range: 29–77). Six participants were male (60%) and four female (40%). Regarding their relationship to the patient, four caregivers were spouses, three were parents, and three were descendants. With respect to employment status, six participants were retired and four were employed. The mean level of education was 11.4 years (SD = 5.3).

### 2.4. Intervention and System Description

The intervention consisted of a semi-immersive VR-based physical training program (K-HERO^®^, CE Class I medical device), administered in a dedicated room within the Day Hospital to ensure standardized conditions and minimize distractions. Each participant completed six supervised sessions lasting 30–40 min during the rehabilitation program (see Table 2).

Training was delivered using the K•One totem, which integrated a high-definition depth-sensing camera and a touch display for real-time interaction. Participants were positioned approximately 2–3 m from the device, and a brief calibration procedure was performed at the beginning of each session to align the participant with the virtual trainer and ensure optimal tracking. Each therapy session followed a standardized sequence: briefing, exercise demonstration, active performance, and a final assessment of mobility, perceived exertion (VAS), and user feedback.

Rehabilitation sessions were structured using predefined motor exercises selected from the K-HERO^®^ library of validated clinical protocols and functional tests. The K•Cloud platform enabled clinicians to configure personalized therapy plans, assign and adapt exercises, and monitor patient performance through a secure dashboard. Body movements were tracked in real time, and mobility indices were automatically computed. These indices, based on cosine similarity between the participant’s performance and a reference model, were expressed on a normalized 0–1 scale and reported separately for the right side, left side, and whole body. Higher scores reflected greater similarity to the reference movement.

These quantitative metrics provided an objective measure of motor accuracy across sessions. Reliability was supported by internal validation, consistent with previously reported reproducibility of depth-sensor-based motion analysis systems. Progression was determined by clinical staff according to performance trends, and all activities were tailored to the participant’s functional capacity. Real-time visual and auditory feedback guided correct execution and enhanced engagement.

### 2.5. Data Governance and Privacy

All motion capture data were processed in real time and stored exclusively in anonymized form on the institutional K•Cloud servers. Each participant was assigned a unique alphanumeric identifier, and no personal identifiers or raw video images were saved. Data were encrypted during transfer and archived in compliance with institutional policy and General Data Protection Regulation (GDPR) standards. Access was restricted to authorized members of the research team through password-protected accounts. Data were retained only for the period required to complete the study analyses (five years) and were then permanently deleted in accordance with institutional policies.

All sessions were facilitated by a movement scientist, who ensured proper execution of motor tasks and provided technical support. Each session was adapted to participant’s functional abilities, preferences, and motivational profiles. In addition, a multidisciplinary team (psychologist, physiotherapist, neurologist, and movement scientist) supervised the intervention and systematically monitored safety, collecting self-reported adverse events at the end of each session and documenting any incidents. No adverse events were reported.

**Figure 1:**
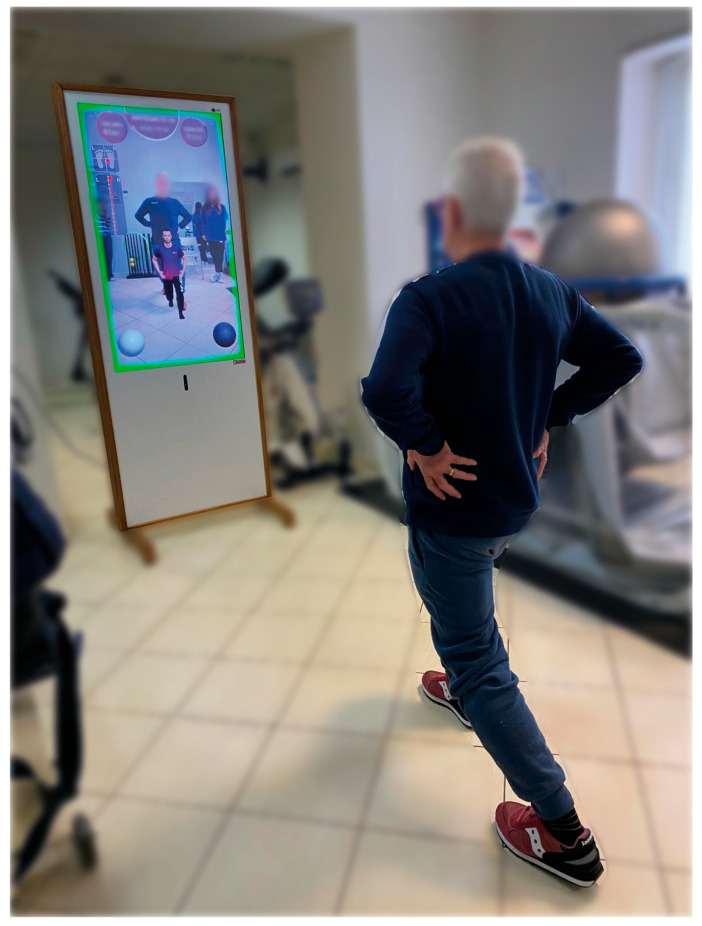
A caregiver during a training session with K-HERO^®^.

K-HERO^®^ is a semi-immersive, markerless VR platform designed to guide and monitor movement without the need for wearable devices (Figure 1). The system employs computer vision and artificial intelligence algorithms to detect anatomical landmarks and track posture, joint angles, and overall mobility in real time. Movement quality was quantified using a cosine similarity algorithm, which compared participants’ skeletal segment angles with those of a digital reference model, providing immediate visual and auditory biofeedback.

The platform’s intuitive interface and engaging graphical environment supported safe and accessible training, even for individuals unfamiliar with technology or exercise routines. Each session combined motor tasks, including reaching, balance training, and functional movements, with a gamified experience to enhance engagement and adherence. The semi-immersive setup allowed caregivers to remain aware of their surroundings while still benefiting from motivating aspects of the digital environment. The system automatically recorded mobility indices (left, right, full) and adherence data, enabling the supervising movement scientist to adjust the program dynamically as needed.

### 2.6. Statistical Analysis

Descriptive statistics were reported as mean ± SD for continuous variables, and as absolute frequencies (percentages) for categorical variables. Age and education were treated as continuous variables, while gender (male, female), relation to patient (spouse, parent, descendant), and employment status/occupation (retired, employee) were analyzed as categorical variables. Clinical and instrumental evaluations at T0 and T1 were expressed as median (first and third quartile) and treated as continuous variables.

Normality of continuous variables was assessed using the Shapiro-Wilk test (MATLAB R2022a, Natick, MA, USA, function swtest). Since most parameters did not follow a normal distribution, non-parametric tests were applied. Correlations between age and the delta (difference between T1 and T0) of clinical and instrumental outcomes were assessed using Spearman’s rank correlation coefficient (corr function, Spearman method, MATLAB).

Differences between T0 and T1 for each outcome were evaluated using the Wilcoxon signed-rank test (signrank, MATLAB). Gender effects were examined by comparing males and females at T0, T1, and delta values with the Wilcoxon rank-sum test (ranksum, MATLAB). The impact of caregiver-patient relationship categories on T0, T1, and delta values was explored with the Kruskal-Wallis test.

Effect sizes for within subject comparison between T0 and T1 were reported as Hodges-Lehmann median differences with corresponding 95% confidence intervals, providing an estimate of both magnitude and precision of the observed changes. Given the feasibility nature of this pilot study and the small sample size, no primary outcome was pre-specified and no correction for multiple comparisons was applied. All analyses should therefore be considered exploratory and hypothesis-generating, with effect sizes and confidence intervals reported alongside *p*-values, to complement significance testing and aid interpretation of the practical relevance of findings.

## 3. Results

A total of twelve participants were initially enrolled in the study. Two individuals discontinued participation due to the interruption of rehabilitation by their associated patients, resulting in a final sample of ten participants. Their main demographic characteristics are summarized in Table 3. The recruitment rate was 100% (12/12). Considering that each participant was scheduled for six sessions (72 sessions in total), the overall session attendance was 89% (64/72 sessions completed). Adherence to the intervention was 100% among participants who completed the program.

**Figure 2:**
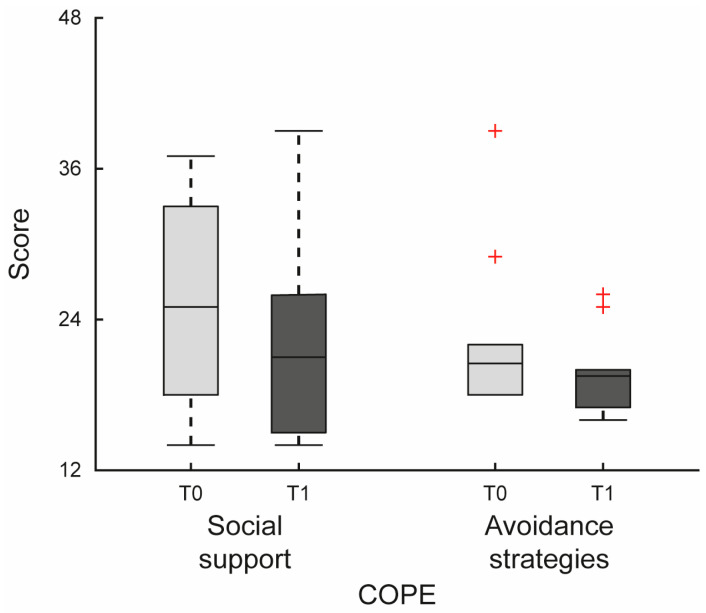
Changes in coping strategies following the intervention. Boxplots represent scores for the COPE subscales Social support and Avoidance strategies at T0 and T1. Red crosses indicate outliers.

A statistically significant difference was observed between T0 and T1 for the COPE subscales Social support (Wilcoxon signed-rank test, *p* = 0.023; Hodges-Lehmann median difference = −3.5, 95% CI [−6, 0]) and Avoidance strategies (*p* = 0.033; Hodges-Lehmann median difference = −3, 95% CI [−3.5, 0.5]). Although statistically significant, both confidence intervals included zero, suggesting uncertainty regarding the true magnitude of the effect. These findings should therefore be interpreted with caution given the small sample size (see Figure 2 and Table 4).

**Figure 3:**
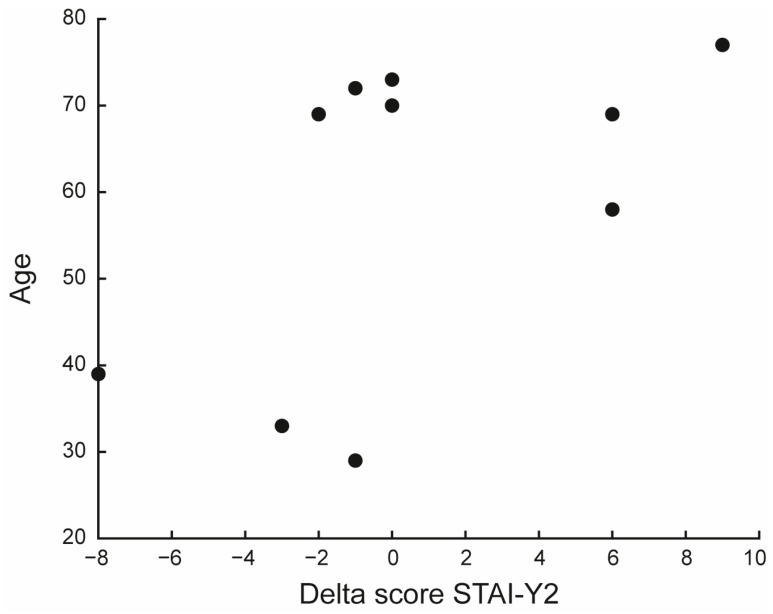
Correlation between age and change in State-Trait Anxiety Inventory—Trait subscale (STAI-Y2) scores. The figure shows a scatter plot displaying the delta score for each participant.

A positive but non-significant correlation was found between age and the delta scores of the STAI-Y2 subscale (ρ = 0.568, *p* = 0.087), indicating that older caregivers may exhibit greater changes over time. This trend warrants further investigation with larger samples (see Figure 3 and Table S1).

**Figure 4:**
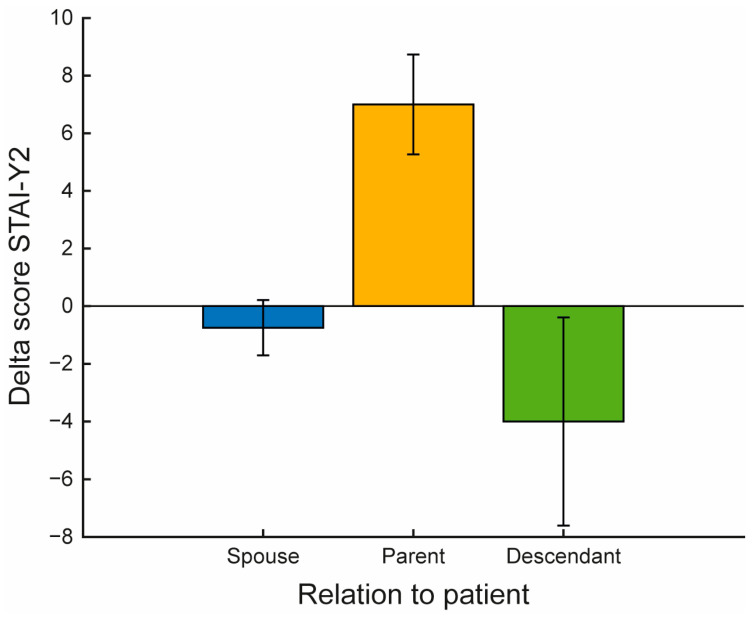
Differences in State-Trait Anxiety Inventory—Trait subscale (STAI-Y2) delta scores among caregivers based on their relationship to the patient. The figure shows a bar plot representing the mean ± SD of the delta scores (T1 − T0) for each group of caregivers, stratified according to their relationship to the patient (spouse, parent, descendant).

When stratified by relationship to the patient, a statistically significant difference was found in the delta scores of the STAI-Y2 subscale (*p* = 0.028) (see Table S2). Specifically, the mean ± SD of −0.75 ± 0.96 for spouses, 7 ± 1.73 for parents, and −4 ± 3.61 for descendants (see Figure 4).

**Figure 5:**
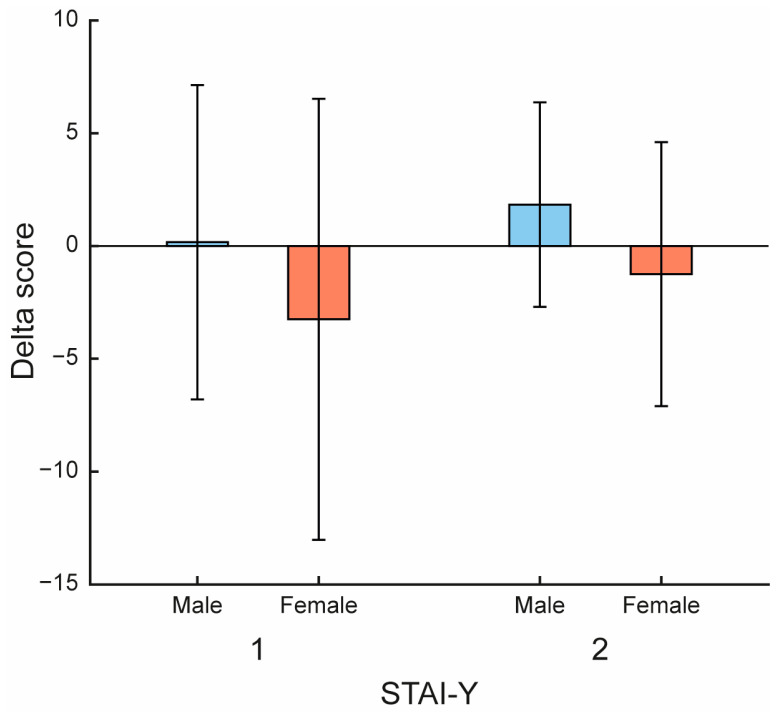
Gender differences in delta scores. Bar plots show mean ± SD for the State-Trait Anxiety Inventory—State subscale (STAI-Y1) and Trait subscale (STAI-Y2).

A gender effect was observed at T0 for both STAI-Y1 (*p* = 0.048) and STAI-Y2 (*p* = 0.029), which was no longer present at T1 (*p* = 0.190) (see Table S3). This pattern reflects a greater increase at T1 in women, resulting in lower delta scores compared with men. Specifically, the delta score (mean ± SD) for STAI-Y1 was −3.25 ± 9.78 in women and 0.17 ± 6.97 in men, while for STAI-Y2 it was −1.25 ± 5.85 in women and 1.83 ± 4.54 in men (see Figure 5).

**Figure 6:**
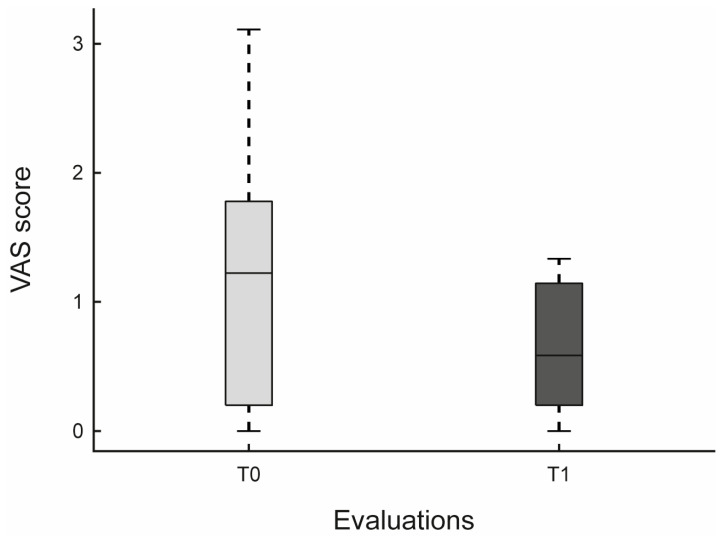
Comparison of Visual Analogue Scale (VAS) scores. Boxplots represent median, interquartile range, and minimum/maximum values at T0 and T1.

An indicative reduction in VAS scores at T1 compared with T0 was also observed (*p* = 0.065). Although the *p*-value did not meet the conventional threshold for significance, the Hodges-Lehmann median difference was −0.656 (95% CI [−1.333, −0.151]), suggesting a likely negative difference and supporting the possibility of a meaningful reduction in perceived pain (see Figure 6 and Table 4).

With respect to instrumental measures, a significant gender difference was found for Mobility Full at T0 (*p* = 0.038), which was no longer evident at T1 (*p* = 0.257) (see Table S3). This finding was driven by a reduction in mobility at T1 among women, as indicated by the delta score (mean ± SD) of −0.07 ± 0.12 for women versus 0.08 ± 0.06 for men. Additional gender differences were observed for Mobility Left at baseline (T0, *p* = 0.019) and for the delta score (T1 − T0, *p* = 0.038). Similarly, a significant gender effect emerged for the Mobility Full delta (T1 − T0, *p* = 0.038), further highlighting baseline and change scores in mobility indices between male and female caregivers (see Table S3).

## 4. Discussion

This exploratory pilot study investigated the effect of a semi-immersive, VR-based physical training program on psychological well-being and coping strategies in informal caregivers of MCI patients. delivered during caregivers’ waiting periods in a rehabilitation facility, the intervention was designed to reduce stress reduction and encourage self-care through a scalable and time-efficient format. While the short duration (six sessions) and the small sample limit the generalizability of findings, the results provide preliminary evidence and highlight promising directions for future research and clinical application.

The most relevant and statistically significant findings concern the reduction in maladaptive coping strategies, particularly avoidance. As measured by the COPE Inventory, informal caregivers who underwent semi-immersive, VR-based intervention showed a significant decrease in their tendency to adopt avoidant behaviors [38,39]. This reduction suggests a positive shift toward more adaptive stress management, potentially mediated by increased body awareness and sensorimotor engagement through the VR experience. These findings support existing evidence that brief interventions can modulate maladaptive patterns even over short timeframes. Avoidance, widely recognized in the literature as a maladaptive coping mechanism associated with poor psychological outcomes, chronic stress, and caregiver burnout, may thus be reduced when integrated into routines and supported by technology-enhanced feedback [40,41].

Interpreting the COPE findings, the observed reduction in social support-based coping could reflect greater autonomy and self-efficacy in managing caregiving demands, as reported in previous research [42,43]. In any case, alternative explanations should also be considered. These include reduced help-seeking or a diminished perception of available support, which could be detrimental. Because no direct measures of self-efficacy or related constructs were collected, such interpretations remain speculative. Prior studies have linked greater internal locus of control and higher self-efficacy to reduced reliance on external validation [44], yet our data are insufficient to draw firm conclusions. 

Similarly, subgroup analyses by caregiver relationship, gender, and age should be interpreted with caution because of the limited sample size and lack of statistical power. These results should therefore be considered exploratory and hypothesis-generating, with effect sizes and confidence intervals reported to enhance transparency about the magnitude and variability of the observed differences.

The observed decline in social support coping should not be construed as inherently beneficial and warrants cautious interpretation within the broader caregiving context. While social support remains vital, further research is needed to clarify how its decline should be understood in interventions that aim to promote autonomy and agency.

Regarding perceived stress (PSS), no statistically significant differences were observed between baseline and post-intervention assessments (median 16 vs. 12, *p* = 0.426). Although the median values suggest a numerical decrease, this trend should be interpreted cautiously due to the absence of statistical significance and the small sample size. Future studies with larger samples are needed to clarify whether VR-based interventions may influence perceived stress in caregivers. Given that chronic stress in caregivers is linked to deterioration in physical and mental health [4], even moderate reductions is consistent with previous literature, highlighting the value of brief self-care interventions for caregivers [45], particularly when implemented during unstructured and emotionally demanding periods.

Another notable finding is illustrated in Figure 4, which highlights differential psychological responses based on the relationship between caregiver and patient. Parents showed a marked increase in psychological distress (STAI-Y2 outcome) following the intervention, whereas offspring (e.g., adult children or grandchildren) showed a decrease, and spouses remained relatively unchanged. Although the sample size limits statistical inference, these trends suggest potentially meaningful relational dynamics. These differences may reflect variations in emotional proximity, caregiving expectations, and role perception. For offspring, the VR-based activity may have provided a rare opportunity for emotional detachment and self-regulation, leading to the observed reduction in trait anxiety. This is consistent with findings suggesting that structured interventions can attenuate secondary stress in younger caregivers by restoring a sense of personal space and self-efficacy [46]. In contrast, the increase in anxiety observed in parental caregivers may indicate heightened awareness of their child’s cognitive decline or unmet emotional needs, brought into focus during the intervention. This may be particularly relevant in neurodegenerative diseases, where role reversal (i.e., parents caring for adult children) often intensifies emotional tension and existential distress [47]. While parental caregivers are less commonly represented in studies on cognitive impairment, their presence in our sample likely reflects the inclusion of relatively young patients (aged 40 and above), making parental caregiving possible. This finding underscores the need to account for diverse caregiver-patient dynamics when designing and implementing supportive interventions. In this subgroup, brief physical interventions alone may not be sufficient and should ideally be complemented by more structured psychological support, helping process feelings of guilt, grief, or anticipated loss. Further research is needed to quantify the extent of these relational effects and assess whether targeted modules, perhaps incorporating mindfulness or emotional reframing, can improve outcomes for caregivers with greater emotional vulnerability.

Gender-related patterns also appeared in our findings. As shown in Figure 5, female caregivers experienced greater reductions in both state (STAI-Y1) and trait (STAI-Y2) anxiety compared with males, with a mean delta of −3.25 for state anxiety and −1.25 for trait anxiety. While these changes are modest, they suggest a potential trend toward gender-related differences in responsiveness to the intervention, particularly in the context of non-pharmacological strategies. These findings support the value of gender-sensitive approaches in caregiver support design, particularly when integrating digital tools. Our results are consistent with previous findings indicating that women tend to report higher baseline anxiety levels but also demonstrate greater responsiveness to structured psychological and physical interventions, including VR-based treatments [1,2]. This trend may be linked to more proactive emotional engagement and a greater openness to seeking support, a profile well suited to the embodied, feedback-rich nature of VR environments. Interestingly, male caregivers in our sample showed no improvement, or even slight worsening, in anxiety scores, particularly for trait anxiety. This could reflect increased emotional awareness or cognitive confrontation triggered by the intervention, as suggested in prior studies on male caregivers who tend to suppress emotional expression and perceive caregiving as a challenge to autonomy or control [48,49]. The VR setting, which promotes body-based awareness and focus, may have paradoxically enhanced their contact with distress, at least initially. Similar gender differences in response to immersive technologies have been reported in neurorehabilitation trials, where women tend to report higher perceived usefulness, lower technophobia, and greater motivation [50].

Moreover, a positive association between age and improvement in STAI-Y2 psychological outcome was found, indicating that older caregivers tended to benefit more from VR-based intervention than their younger counterparts, as shown in Figure 3. Although exploratory, this trend is consistent with previous evidence suggesting that age is positively related to baseline anxiety, particularly in caregiving settings, which may reflect reduced cognitive flexibility, heightened awareness of functional decline, and cumulative emotional burden [51]. From a clinical perspective, this pattern supports the idea that older adults may be particularly responsive to structured, low-demand interventions, that provide embodied feedback, gentle activation, and psychological support. Interestingly, despite often being perceived as “digitally excluded,” older caregivers in our sample interacted effectively with the VR system, likely facilitated by its intuitive interface and markerless design. This finding align with growing evidence showing that, when technological barriers are minimized, older adults are not only willing but also able to benefit from digital health tools [52,53]. Furthermore, older caregivers are often chronically exposed to stressors, including prolonged caregiving, social isolation, and physical fatigue [51]. Therefore, even moderate psychological improvements in this population could have substantial clinical relevance by helping prevent burnout and comorbidities. The positive age-outcome correlation observed in this study suggests that this subgroup could be prioritized in future targeted interventions, with a focus on personalization, appropriate pacing, and emotional accessibility.

Finally, aspects related to pain perception and mobility should be further considered, given their potential clinical implications. An apparent pain reduction was observed at post-intervention on Visual Analogue Scale (VAS). Although not statistically significant (*p* = 0.065), this change suggests a possible improvement in subjective well-being, potentially mediated by engagement in physical activity and psychosocial stimulation. Previous studies have shown that structured physical activity programs, even when mediated by technology, can modulate pain perception through both physiological (e.g., endorphin release, improved circulation) and psychological (e.g., attention distraction, increased self-efficacy) mechanisms [54].

### Limitation of the Study and Clinical Application

Although promising, this study has several limitations. The small and non-randomized sample limits the generalizability of the findings. It also increases the risk of type II error, particularly for outcomes that showed clinically meaningful changes but did not reach statistical significance. Moreover, the small sample size and the absence of a control group preclude causal inference. Given the absence of a pre-specified primary endpoint and adjustment for multiplicity, the findings remain exploratory and hypothesis-generating rather than confirmatory. The short duration of the intervention (six sessions), while practical, may not be adequate to produce lasting psychological change, especially in caregivers of patients suffering from neurodegenerative diseases. Moreover, the effects of VR intervention were not compared with conventional physical exercises or alternative activities that could provide comparable or greater benefits. Furthermore, the use of a validated self-report measures remains subject to bias, especially in emotionally vulnerable populations. Moreover, the overrepresentation of male caregivers (60%), although unusual and informative, may have generated gender-specific effects, restricting comparability with previous studies predominantly focused on female caregivers. Finally, it remains unclear whether the assessed parameters in caregivers differ from those observed in the general population, further limiting generalizability.

From a clinical perspective, this pilot study could suggest that in situ digital micro-interventions like K-HERO^®^ as scalable and inclusive tools to support caregiver well-being. The intervention was well tolerated even among older and less educated individuals, indicating that limited technological literacy may not be a barrier when using an intuitive, user-centered system. Notably, tailoring intervention strategies to the caregiver characteristics such as age, gender, and relationship with the patient, can improve engagement and effectiveness of digital interventions. These findings could support the development of personalized digital interventions, particularly for caregivers who may otherwise lack the time, access, or motivation to access traditional psychological support.

## 5. Conclusions

Overall, our findings support the feasibility and potential utility of brief VR-based interventions for informal caregivers, particularly during waiting times in clinical settings. The data could suggest that even low-intensity programs can promote positive changes in coping strategies, reduce avoidant behaviors, and support emotional well-being. These effects appear influenced by relational as well as age- and gender-related factors. However, the findings should be interpreted with caution due to the small sample size and exploratory design. Future research should focus on large-scale, controlled trials to confirm these findings. It should also explore the long-term impact of such interventions. Finally, the inclusion of qualitative data may offer a better understanding of the lived experience and emotional trajectories across caregiver subgroups.

## Figures and Tables

**Table 1:** Summary of Assessment Tools. The table shows the standardized instruments used to assess psychological well-being, perceived stress, coping strategies, physical activity levels, caregiver burden, general health perception, and digital system usability. For each instrument, a brief description of its purpose is provided, along with clinical cut-off scores where available.

Instrument	Description	Clinical Cut-off
**State-Trait Anxiety Inventory (STAI) [30]**	Measures both transient (state) and enduring (trait) anxiety symptoms	>40 indicates clinically relevant anxiety (varies by age/gender)
**Coping Orientation to Problems Experienced (COPE) [31]**	Assesses a wide range of coping strategies (e.g., social support, avoidance, problem-solving)	No specific cut-off; scores analyzed by subscale
**Perceived Stress Scale (PSS) [32]**	Evaluates perceived stress over the past month	≥20 suggests high perceived stress
**International Physical Activity Questionnaire** **(IPAQ-SF) [33]**	Assesses weekly physical activity including walking, moderate, and vigorous exercise	<600 Metabolic Equivalent of Task minutes per week (MET-min/week) = low activity
**Caregiver Burden Inventory (CBI) [34]**	Measures caregiver burden across five dimensions (time-dependence, developmental, physical, social, emotional)	≥24 indicates clinically significant burden
**Beck Depression Inventory-II (BDI-II) [35]**	Self-report inventory assessing depressive symptoms	14–19 mild; 20–28 moderate; 29–63 severe
**Short Form Health Status Survey-12 (SF-12) [36]**	Self-report questionnaire assessing perceived physical and mental health status	>50 indicates better than average physical and health functioning
**System Usability Scale (SUS) [37]**	Assesses user satisfaction and usability of digital systems	>68 acceptable usability; >80 excellent usability

**Table 2:** K-HERO^®^ exercise routine overview.

Section	Description	Duration
Warm-up (stretching and mobility exercises)	Forward torso stretches with arms extension Shoulder and trunk extension, where the arms open outward and the torso rotates right and left, allowing one hand to approach the opposite hand Shoulder mobility exercises, involving alternating upward stretching of the arms Neck extension forward and backward, achieved by gently supporting the cervical spine with the hands	5–10 min
Work-out (posture, mobility, strength, and balance)	Exercises to improve static and dynamic balance Postural exercises targeting the posterior kinetic chain, performed using a stick for alignment support Range of motion exercises to enhance flexibility and mobility in the knees, neck, torso, and shoulders Muscle strengthening exercises, using weights and involving both upper and lower limbs	20–25 min
Cool-down (relaxation and decompression exercises)	Back extension, performed by placing the hands behind the pelvis, pushing it forward, and tilting the head back to open the chest and shoulders Forward head extension, executed while seated by tilting the head downward and applying light pressure with the hands-on top of the head Backward head extension, also from a seated position, where the head is gently extended backward with support from the hands on the back of the neck	5 min

**Table 3:** Demographic characteristics of participants (N = 10). The table shows the mean and standard deviation for participants’ age and years of education. The distribution of gender is expressed as percentage of males and females. The percentage of participants belonging to each category of relationship to the patient (spouse, parent, descendant) is also presented.

Characteristic	
**Age**	
mean ± SD	58.9 ± 18.2
min, max	29, 77
**Gender**	
Male (%)	6 (60%)
Female (%)	4 (40%)
**Education Level**	
mean ± SD	11.4 ± 5.3
**Relationship with patient**	
Spouse (%)	4 (40%)
Parent (%)	3 (30%)
Descendant (%)	3 (30%)
**Employment Status**	
Retired (%)	6 (60%)
Employee (%)	4 (40%)

**Table 4:** Pre- and post-intervention scores on psychological, coping, and physical activity measures in caregivers. Comparison of caregiver outcomes at T0 and T1. Values for T0 and T1 are reported as median (1st–3rd quartile). The reported *p*-values refer to the Wilcoxon signed-rank test used to assess differences between T0 and T1. Effect sizes are presented as Hodges-Lehmann median differences with corresponding 95% confidence intervals to indicate the magnitude and precision of change. *p*-values are reported without correction for multiple comparisons.

	Clinical Scale	T0	T1	*p*-Value	Hodges-Lehmann Median Diff	95% CI
	PSS	16 (11–23)	12 (10–18)	0.426	−1	[−9, 2]
STAI-Y	1	36 (32–46)	40.5 (26–48)	0.590	−2.5	[−7, 5]
	2	35 (32–46)	37 (31–45)	0.930	−0.5	[−2.5, 6]
	CBI	13.5 (9–23)	15 (12–28)	0.414	1	[−2, 6]
	BDI-II	7 (2–13)	9 (1–12)	0.906	0	[−2.5, 1.5]
	SF-12	33.5 (30–34)	33 (32–34)	0.535	−0.5	[−2, 3]
COPE	Social Support	25 (18–33)	21 (15–26)	**0.023**	−3.5	[−6, 0]
	Avoidance Strategies	20.5 (18–22)	19.5 (17–20)	**0.033**	−3	[−3.5, 0.5]
	Positive Attitude	31.5 (28–34)	30.5 (27–33)	0.867	0	[−4.5, 3]
	Orientation Problem	34.5 (31–36)	32.5 (29–38)	0.945	0	[−4, 4]
	Transcendent Orientation	25 (22–27)	22 (20–30)	0.992	−0.5	[−2.5, 3]
IPAQ	Vigorous IPAQ	3660 (0–5760)	1800 (960–4320)	0.391	0	[−2880, 1080]
	Moderate-IPAQ	1320 (0–3360)	1320 (480–5040)	0.723	480.0	[−1560, 1440]
	Walking-IPAQ	478.5 (0–1188)	643.5 (396–1478.4)	0.900	346.5	[−1930.5, 2277]
	IPAQ	6714 (2457–10,764)	5310 (2259–9198)	1.000	2395.2	[−6129, 4158]
	VAS	1.3 (1.27–1.27)	0.6 (0.61–0.61)	0.065	−0.656	[−1.333, −0.151]
	Execution difficulty	0.6 (0.6–0.6)	0.4 (0.4–0.4)	0.813	0	[−1, 0]
	Muscular difficulty	0.4 (0.4–0.4)	0.4 (0.4–0.4)	0.703	0	[−1, 1]
	Balance difficulty	1.4 (1.4–1.4)	0.9 (0.9–0.9)	0.453	−1	[−2, 1]
	**Instrumental data**					
	Mobility left	0.8 (0.78–0.78)	0.8 (0.81–0.81)	0.275	0.051	[−0.017, 0.107]
	Mobility right	0.8 (0.82–0.82)	0.8 (0.85–0.85)	0.160	0.049	[−0.014, 0.080]
	Mobility full	0.7 (0.7–0.7)	0.7 (0.72–0.72)	0.432	0.032	[−0.047, 0.090]

**Legend:** BDI-II = Beck Depression Inventory-II; CBI = Caregiver Burden Inventory; COPE = Coping Orientation to Problems Experienced; IPAQ = International Physical Activity Questionnaire; PSS = Perceived Stress Scale; STAI-Y1 = State-Trait Anxiety Inventory—State Anxiety; STAI-Y2 = State-Trait Anxiety Inventory—Trait Anxiety; SF-12 = 12-Item Short Form Health Survey. Statistically significant *p*-values (*p* < 0.05) are in bold.

## Data Availability

Data presented in this study are available on request from the corresponding author. The data are not publicly available due to the privacy of research participants.

## Supplementary Materials

The following supporting information can be downloaded at: https://www.mdpi.com/article/10.3390/jfmk10030353/s1, Supplementary Material S1: TREND Checklist; Supplementary Figure S1: Flowchart of the patient’s selection process; Supplementary Table S1: Correlation between age and clinical and instrumental outcomes; Supplementary Table S2: Clinical and instrumental outcomes across caregiver groups based on relationship to the patient. Supplementary Table S3: Differences between genders in clinical scales and instrumental measures.

## References

[1] Corallo F., Maggio M.G., Bonanno L., De Luca R., Cardile D., Cappadona I., Todaro A., Calabrò R.S. (2024). Burden in Caregivers of Patients with Acquired Brain Injury: Influence of Family Role and Gender. NeuroRehabilitation.

[2] Maggio M.G., Corallo F., De Francesco M., De Cola M.C., De Luca R., Manuli A., Quartarone A., Rizzo A., Calabrò R.S. (2024). Understanding the Family Burden and Caregiver Role in Stroke Rehabilitation: Insights from a Retrospective Study. Neurol. Sci. Off. J. Ital. Neurol. Soc. Ital. Soc. Clin. Neurophysiol..

[3] Adelman R.D., Tmanova L.L., Delgado D., Dion S., Lachs M.S. (2014). Caregiver Burden: A Clinical Review. JAMA.

[4] Schulz R., Sherwood P.R. (2008). Physical and Mental Health Effects of Family Caregiving. Am. J. Nurs..

[5] Pottie C.G., Burch K.A., Thomas L.P.M., Irwin S.A. (2014). Informal Caregiving of Hospice Patients. J. Palliat. Med..

[6] Sallim A.B., Sayampanathan A.A., Cuttilan A., Ho R.C.-M. (2015). Prevalence of Mental Health Disorders Among Caregivers of Patients With Alzheimer Disease. J. Am. Med. Dir. Assoc..

[7] Capistrant B.D., Moon J.R., Glymour M.M. (2012). Spousal Caregiving and Incident Hypertension. Am. J. Hypertens..

[8] Kiecolt-Glaser J.K., Preacher K.J., MacCallum R.C., Atkinson C., Malarkey W.B., Glaser R. (2003). Chronic Stress and Age-Related Increases in the Proinflammatory Cytokine IL-6. Proc. Natl. Acad. Sci. USA.

[9] von Känel R., Dimsdale J.E., Mills P.J., Ancoli-Israel S., Patterson T.L., Mausbach B.T., Grant I. (2006). Effect of Alzheimer Caregiving Stress and Age on Frailty Markers Interleukin-6, C-Reactive Protein, and D-Dimer. J. Gerontol. Ser. A.

[10] Shulman L.M., Taback R.L., Bean J., Weiner W.J. (2001). Comorbidity of the Nonmotor Symptoms of Parkinson’s Disease. Mov. Disord..

[11] Lawton M., Moss M., Duhamel L. (1995). The Quality of Daily-Life Among Elderly Care Receivers. J. Appl. Gerontol..

[12] Henry R.S., Lageman S.K., Perrin P.B. (2020). The Relationship between Parkinson’s Disease Symptoms and Caregiver Quality of Life. Rehabil. Psychol..

[13] Thornton M., Travis S.S. (2003). Analysis of the Reliability of the Modified Caregiver Strain Index. J. Gerontol. Ser. B.

[14] Bastawrous M. (2013). Caregiver Burden—A Critical Discussion. Int. J. Nurs. Stud..

[15] Greenwood N., Mezey G., Smith R. (2018). Social Exclusion in Adult Informal Carers: A Systematic Narrative Review of the Experiences of Informal Carers of People with Dementia and Mental Illness. Maturitas.

[16] Lopez-Hartmann M., Wens J., Verhoeven V., Remmen R. (2012). The Effect of Caregiver Support Interventions for Informal Caregivers of Community-Dwelling Frail Elderly: A Systematic Review. Int. J. Integr. Care.

[17] Lilly M.B., Robinson C.A., Holtzman S., Bottorff J.L. (2012). Can We Move beyond Burden and Burnout to Support the Health and Wellness of Family Caregivers to Persons with Dementia? Evidence from British Columbia, Canada. Health Soc. Care Community.

[18] Northouse L.L., Katapodi M.C., Song L., Zhang L., Mood D.W. (2010). Interventions with Family Caregivers of Cancer Patients: Meta-Analysis of Randomized Trials. CA. Cancer J. Clin..

[19] Geraets C.N.W., van der Stouwe E.C.D., Pot-Kolder R., Veling W. (2021). Advances in Immersive Virtual Reality Interventions for Mental Disorders: A New Reality?. Curr. Opin. Psychol..

[20] Bruno R.R., Wolff G., Wernly B., Masyuk M., Piayda K., Leaver S., Erkens R., Oehler D., Afzal S., Heidari H. (2022). Virtual and Augmented Reality in Critical Care Medicine: The Patient’s, Clinician’s, and Researcher’s Perspective. Crit. Care.

[21] Kanschik D., Bruno R.R., Wolff G., Kelm M., Jung C. (2023). Virtual and Augmented Reality in Intensive Care Medicine: A Systematic Review. Ann. Intensive Care.

[22] Jung C., Wolff G., Wernly B., Bruno R.R., Franz M., Schulze P.C., Silva J.N.A., Silva J.R., Bhatt D.L., Kelm M. (2022). Virtual and Augmented Reality in Cardiovascular Care: State-of-the-Art and Future Perspectives. JACC Cardiovasc. Imaging.

[23] Kundu M., Ng J.C., Awuah W.A., Huang H., Yarlagadda R., Mehta A., Nansubuga E.P., Jiffry R., Abdul-Rahman T., Ou Yong B.M. (2023). NeuroVerse: Neurosurgery in the Era of Metaverse and Other Technological Breakthroughs. Postgrad. Med. J..

[24] Wolff G., Bruno R.R., Reiter M., Kantzow B., Kelm M., Jung C. (2020). Virtual Reality Device Training for Extracorporeal Membrane Oxygenation. Crit. Care.

[25] Sutherland J., Belec J., Sheikh A., Chepelev L., Althobaity W., Chow B.J.W., Mitsouras D., Christensen A., Rybicki F.J., La Russa D.J. (2019). Applying Modern Virtual and Augmented Reality Technologies to Medical Images and Models. J. Digit. Imaging.

[26] Hoffman H.G., Rodriguez R.A., Gonzalez M., Bernardy M., Peña R., Beck W., Patterson D.R., Meyer W.J. (2019). Immersive Virtual Reality as an Adjunctive Non-Opioid Analgesic for Pre-Dominantly Latin American Children with Large Severe Burn Wounds During Burn Wound Cleaning in the Intensive Care Unit: A Pilot Study. Front. Hum. Neurosci..

[27] Puel F., Minville V., Vardon-Bounes F. (2021). What Place for Virtual Reality in the Intensive Care Unit during Medical Procedures?. J. Intensive Care.

[28] Silva J.N.A., Southworth M., Raptis C., Silva J. (2018). Emerging Applications of Virtual Reality in Cardiovascular Medicine. JACC Basic Transl. Sci..

[29] Des Jarlais D.C., Lyles C., Crepaz N., the TREND Group (2004). Improving the Reporting Quality of Nonrandomized Evaluations of Behavioral and Public Health Interventions: The TREND Statement. Am. J. Public Health.

[30] Marteau T.M., Bekker H. (1992). The Development of a Six-Item Short-Form of the State Scale of the Spielberger State—Trait Anxiety Inventory (STAI). Br. J. Clin. Psychol..

[31] Cope S.M., Liu X.-C., Verber M.D., Cayo C., Rao S., Tassone J.C. (2010). Upper Limb Function and Brain Reorganization after Constraint-Induced Movement Therapy in Children with Hemiplegia. Dev. Neurorehabilit..

[32] Cohen S., Kamarck T., Mermelstein R. (1983). A Global Measure of Perceived Stress. J. Health Soc. Behav..

[33] Washburn R.A., Montoye H.J. (1986). The assessment of physical activity by questionnaire. Am. J. Epidemiol..

[34] Novak I., Morgan C., Fahey M., Finch-Edmondson M., Galea C., Hines A., Langdon K., Namara M.M., Paton M.C., Popat H. (2020). State of the Evidence Traffic Lights 2019: Systematic Review of Interventions for Preventing and Treating Children with Cerebral Palsy. Curr. Neurol. Neurosci. Rep..

[35] Beck A.T., Steer R.A., Brown G. (2011). Beck Depression Inventory–II 2011.

[36] Kodraliu G., Mosconi P., Groth N., Carmosino G., Perilli A., Gianicolo E.A., Rossi C., Apolone G. (2001). Subjective Health Status Assessment: Evaluation of the Italian Version of the SF-12 Health Survey. Results from the MiOS Project. J. Epidemiol. Biostat..

[37] Finstad K. (2010). The Usability Metric for User Experience. Interact. Comput..

[38] Dilgul M., Martinez J., Laxhman N., Priebe S., Bird V. (2020). Cognitive Behavioural Therapy in Virtual Reality Treatments across Mental Health Conditions: A Systematic Review. Consort. Psychiatr..

[39] Cáceres-Matos R., Castillo-García M., Magni E., Pabón-Carrasco M. (2024). Effectiveness of Virtual Reality for Managing Pain, Fear, and Anxiety in Children and Adolescents Undergoing Needle-Related Procedures: Systematic Review with Meta-Analysis. Nurs. Rep..

[40] Meurer K.J., Presciutti A.M., Bannon S.M., Kubota R., Baskaran N., Kim J., Zhang Q., Reichman M., Fishbein N.S., Lichstein K. (2025). Characterizing Stressors and Coping Strategies Among Caregivers of Patients with Severe Acute Brain Injury by Level of Distress. Neurocrit. Care.

[41] Schoenmakers B., Buntinx F., De Lepeleire J. (2009). The Relation between Care Giving and the Mental Health of Caregivers of Demented Relatives: A Cross-Sectional Study. Eur. J. Gen. Pract..

[42] Wang J., Li Q., Cui J., Tu S., Deng Z., Yang R., Wang Y. (2023). Effectiveness of Virtual Reality on the Caregiving Competence and Empathy of Caregivers for Elderly with Chronic Diseases: A Systematic Review and Meta-Analysis. J. Nurs. Manag..

[43] Li Pira G., Aquilini B., Davoli A., Grandi S., Ruini C. (2023). The Use of Virtual Reality Interventions to Promote Positive Mental Health: Systematic Literature Review. JMIR Ment. Health.

[44] Botha F., Dahmann S.C. (2024). Locus of Control, Self-Control, and Health Outcomes. SSM-Popul. Health.

[45] Collins S.P., Storrow A., Liu D., Jenkins C.A., Miller K.F., Kampe C., Butler J. (2021). Examining Whether a Self-Care Program Reduces Healthcare Use and Improves Health Among Patients with Acute Heart Failure—The Guided HF Study.

[46] Ko E., Wongvibul T., Rose K.M., Jun J. (2023). The Effects of Self-Guided Interventions on Stress, Burden, and Mental Health in Caregivers of People Living with Dementia: A Systematic Review. Int. J. Nurs. Stud. Adv..

[47] Habermann B., Hines D., Davis L. (2013). Caring for Parents with Neurodegenerative Disease: A Qualitative Description. Clin. Nurse Spec. CNS.

[48] Khalaila R., Cohen M. (2016). Emotional Suppression, Caregiving Burden, Mastery, Coping Strategies and Mental Health in Spousal Caregivers. Aging Ment. Health.

[49] Keng S.-L., Smoski M.J., Robins C.J. (2011). Effects of Mindfulness on Psychological Health: A Review of Empirical Studies. Clin. Psychol. Rev..

[50] Bruschetta R., Maggio M.G., Naro A., Ciancarelli I., Morone G., Arcuri F., Tonin P., Tartarisco G., Pioggia G., Cerasa A. (2022). Gender Influences Virtual Reality-Based Recovery of Cognitive Functions in Patients with Traumatic Brain Injury: A Secondary Analysis of a Randomized Clinical Trial. Brain Sci..

[51] Christian L.M., Wilson S.J., Madison A.A., Prakash R.S., Burd C.E., Rosko A.E., Kiecolt-Glaser J.K. (2023). Understanding the Health Effects of Caregiving Stress: New Directions in Molecular Aging. Ageing Res. Rev..

[52] Nikou S., Agahari W., Keijzer-Broers W., De Reuver M. (2020). Digital Healthcare Technology Adoption by Elderly People: A Capability Approach Model. Telemat. Inform..

[53] Bauge K., Babic A. (2025). Gaming for Cognitive Assessment and Enhancement in Elders: A Secondary Analysis of Literature and Applications. Stud. Health Technol. Inform..

[54] Basso J.C., Suzuki W.A. (2017). The Effects of Acute Exercise on Mood, Cognition, Neurophysiology, and Neurochemical Pathways: A Review. Brain Plast..

