# Supplementary material for: Low-Intensity Virtual Reality Exercise for Caregivers of People with Mild Cognitive Impairment: A Pilot Study
Source: J Funct Morphol Kinesiol. 2025 Sep 16;10(3):353. doi: 10.3390/jfmk10030353 (PMC12452558; doi:10.3390/jfmk10030353)
Supplement: Supplementary file 1 [file jfmk-10-00353-s001.zip › Table S1.pdf]

Table S1. Correlation between age and clinical and instrumental outcomes. The table reports, for each clinical assessment and instrumental measure, the correlation results with age, including the Spearman's rho coefficient and the corresponding p-value. No correction for multiple comparisons was applied.

	**Clinical scale**	**ϱ corr**	**p-value**
	PSS	0.230	0.523
STAI-Y	1	0.268	0.455
	2	0.568	0.087
	CBI	0.331	0.350
	BDI-II	−0.096	0.792
	SF-12	−0.102	0.779
COPE	Social Support	−0.499	0.143
	Avoidance Strategies	−0.543	0.105
	Positive Attitude	−0.095	0.794
	Orientation Problem	0.150	0.679
	Transcendent Orientation	−0.223	0.535
IPAQ	Vigorous IPAQ	0.142	0.697
	Moderate-IPAQ	0.122	0.737
	Walking-IPAQ	0.083	0.821
	IPAQ	0.304	0.393
	SUS	0.049	0.893
	VAS	0.103	0.776
	Execution difficulty	0.179	0.621
	Muscular difficulty	0.398	0.255
	Balance difficulty	0.378	0.282
	**Instrumental data**		
	Mobility left	0.310	0.383
	Mobility right	0.280	0.434
	Mobility full	0.450	0.192
