# Supplementary material for: Low-Intensity Virtual Reality Exercise for Caregivers of People with Mild Cognitive Impairment: A Pilot Study
Source: J Funct Morphol Kinesiol. 2025 Sep 16;10(3):353. doi: 10.3390/jfmk10030353 (PMC12452558; doi:10.3390/jfmk10030353)
Supplement: Supplementary file 1 [file jfmk-10-00353-s001.zip › Table S2.pdf]

Table S2. Clinical and instrumental outcomes across caregiver groups based on relationship to the patient (Spouse, Parent, Descendant). The table reports clinical scale scores and instrumental measures stratified by caregiver relationship to the patient. For each parameter, the table includes the p-values from the Kruskal-Wallis test performed at T0 at T1, and on delta scores to assess differences between groups. Additionally, for each group and each time point (T0, T1, delta), values are presented as median (1st – 3rd quartile). No correction for multiple comparisons was applied.

					Group 1			Group 2			Group 3		
	**Clinical scale**	**p-value T0**	**p-value T1**	**p-value Delta**	**T0**	**T1**	**Delta**	**T0**	**T1**	**Delta**	**T0**	**T1**	**Delta**
	PSS	0.689	0.720	0.266	14.5 (9.0 – 18.0)	15.5 (8.5 – 21.0)	1.5 (−0.5 – 3.0)	15.0 (12.0 – 24.0)	14.0 (11.0 – 17.0)	−1.0 (−7.0 – −1.0)	23.0 (11.8 – 24.5)	10.0 (8.5 – 14.5)	−9.0 (−13.5 – −0.8)
STAI-Y	1	0.471	0.676	0.264	33.5 (29.5 – 44.0)	37.0 (24.0 – 49.5)	−3.5 (−5.5 – 5.5)	40.0 (37.0 – 44.5)	43.0 (39.2 – 48.2)	2.0 (−1.8 – 8.0)	36.0 (33.0 – 51.8)	32.0 (26.0 – 44.0)	−9.0 (−11.2 – −2.2)
	2	0.555	0.299	**0.0280**	32.0 (30.0 – 41.0)	31.5 (28.5 – 41.0)	−0.5 (−1.5 – 0.0)	36.0 (34.5 – 43.5)	42.0 (40.5 – 51.8)	6.0 (6.0 – 8.2)	42.0 (34.5 – 46.5)	34.0 (31.8 – 42.2)	−3.0 (−6.8 – −1.5)
	CBI	0.264	0.208	0.613	16.5 (5.5 – 22.5)	19.0 (7.0 – 28.5)	6.0 (−3.5 – 11.0)	30.0 (15.0 – 31.5)	28.0 (18.2 – 30.2)	−1.0 (−1.8 – 3.5)	9.0 (3.8 – 14.2)	12.0 (3.0 – 14.2)	−1.0 (−1.8 – 2.0)
	BDI-II	0.0920	0.148	0.719	4.5 (2.5 – 7.0)	5.5 (1.0 – 9.0)	0.5 (−1.5 – 2.0)	16.0 (12.2 – 19.8)	12.0 (10.5 – 21.0)	1.0 (−4.2 – 2.5)	2.0 (1.2 – 10.2)	1.0 (0.2 – 10.0)	0.0 (−1.5 – 0.0)
	SF-12	0.367	0.240	0.649	32.0 (25.0 – 34.0)	32.5 (25.5 – 33.0)	−1.0 (−1.5 – 1.0)	31.0 (30.2 – 33.2)	33.0 (30.0 – 36.8)	3.0 (−0.8 – 3.8)	34.0 (33.2 – 34.8)	34.0 (33.2 – 34.8)	0.0 (−1.5 – 1.5)
COPE	Social Support	0.617	0.321	0.633	21.0 (16.0 – 29.0)	17.5 (14.5 – 21.0)	−4.0 (−8.0 – −1.5)	26.0 (23.0 – 31.2)	25.0 (21.2 – 25.8)	−2.0 (−6.5 – −0.5)	30.0 (21.0 – 35.2)	27.0 (17.2 – 36.0)	−3.0 (−3.8 – 0.8)
	Avoidance Strategies	0.381	0.430	0.259	21.5 (19.5 – 25.5)	20.0 (19.5 – 22.5)	−2.0 (−3.5 – 0.5)	20.0 (19.2 – 34.2)	17.0 (16.2 – 23.8)	−3.0 (−10.5 – −3.0)	18.0 (18.0 – 20.2)	18.0 (17.2 – 19.5)	−1.0 (−2.5 – 1.2)
	Positive Attitude	0.819	0.523	0.402	30.5 (21.5 – 36.0)	34.5 (23.0 – 38.5)	1.5 (−0.5 – 4.5)	31.0 (30.2 – 38.5)	31.0 (26.5 – 32.5)	0.0 (−12.0 – 2.2)	32.0 (29.0 – 33.5)	29.0 (27.5 – 29.8)	−2.0 (−5.8 – 0.2)
	Orientation Problem	0.190	0.943	0.206	32.5 (21.5 – 34.5)	35.0 (22.0 – 38.5)	2.5 (0.5 – 4.0)	36.0 (34.5 – 36.8)	33.0 (24.0 – 36.0)	−1.0 (−11.5 – −0.2)	36.0 (32.2 – 36.0)	30.0 (29.2 – 38.2)	−1.0 (−5.5 – 3.5)
	Transcendent Orientation	0.584	0.788	0.389	26.5 (22.0 – 28.0)	22.5 (20.5 – 28.0)	0.0 (−6.0 – 4.5)	23.0 (22.2 – 23.8)	22.0 (20.5 – 22.0)	−2.0 (−2.0 – −1.2)	26.0 (20.0 – 29.8)	30.0 (21.0 – 30.8)	0.0 (0.0 – 3.0)
IPAQ	Vigorous-IPAQ	0.722	0.992	0.699	3600.0 (720.0 – 7920.0)	2640.0 (480.0 – 7200.0)	0.0 (−2400.0 – 1440.0)	0.0 (0.0 – 5400.0)	2160.0 (540.0 – 4860.0)	0.0 (−1080.0 – 1620.0)	4320.0 (3330.0 – 4680.0)	1440.0 (1440.0 – 3240.0)	−2880.0 (−3240.0 – −90.0)

Moderate-IPAQ	0.577	0.649	0.331	1080.0 (360.0 – 2400.0)	1320.0 (240.0 – 3600.0)	960.0 (−480.0 – 1560.0)	0.0 (0.0 – 3780.0)	5040.0 (1620.0 – 5040.0)	480.0 (120.0 – 3900.0)	2400.0 (1500.0 – 3840.0)	720.0 (720.0 – 1620.0)	−1680.0 (−3120.0 – 120.0)
Walking-IPAQ	0.247	0.840	0.794	148.5 (0.0 – 1930.5)	643.5 (495.0 – 2425.5)	346.5 (−1287.0 – 2277.0)	0.0 (0.0 – 891.0)	396.0 (99.0 – 3217.5)	396.0 (−792.0 – 3217.5)	1188.0 (792.0 – 3415.5)	1386.0 (420.8 – 1455.3)	−1089.0 (−2351.2 – 341.6)
IPAQ	0.674	0.588	0.220	6610.5 (1228.5 – 12102.0)	4363.5 (1264.5 – 13176.0)	2746.5 (−4117.5 – 5227.5)	5040.0 (1260.0 – 7551.0)	9198.0 (4576.5 – 10399.5)	3036.0 (2568.0 – 3877.5)	8388.0 (5742.0 – 11695.5)	3546.0 (2580.8 – 6315.3)	−6129.0 (−8471.2 – 251.6)
SUS	0.787			77.5 (68.8 – 91.2)			80.0 (70.6 – 81.9)			85.0 (66.2 – 92.5)		
VAS	0.849	0.624	0.705	1.1 (0.5 – 2.1)	0.5 (0.2 – 0.9)	−0.7 (−1.4 – −0.1)	1.3 (0.3 – 1.7)	1.3 (0.3 – 1.3)	−0.4 (−1.1 – 0.8)	1.7 (0.6 – 2.3)	0.6 (0.1 – 0.6)	−1.1 (−1.6 – −0.4)
Execution difficulty	0.230	0.565	0.892	0.5 (0.0 – 1.0)	0.0 (0.0 – 0.5)	−0.5 (−1.0 – 0.5)	1.0 (0.2 – 2.5)	0.0 (0.0 – 2.2)	0.0 (−2.2 – 1.5)	0.0 (0.0 – 0.0)	0.0 (0.0 – 0.0)	0.0 (0.0 – 0.0)
Muscular difficulty	1	0.444	0.776	0.0 (0.0 – 1.0)	0.5 (0.0 – 1.0)	0.5 (−1.0 – 1.0)	0.0 (0.0 – 0.8)	0.0 (0.0 – 1.5)	0.0 (−0.8 – 1.5)	0.0 (0.0 – 0.8)	0.0 (0.0 – 0.0)	0.0 (−0.8 – 0.0)
Balance difficulty	0.933	0.213	0.492	1.5 (0.5 – 2.0)	0.5 (0.0 – 1.5)	−1.0 (−1.5 – 0.5)	1.0 (1.0 – 1.8)	2.0 (0.5 – 3.5)	1.0 (−1.2 – 2.5)	1.0 (1.0 – 2.5)	0.0 (0.0 – 0.0)	−1.0 (−2.5 – −1.0)
**Instrumental data**												
Mobility left	0.102	0.943	0.317	0.7 (0.7 – 0.7)	0.8 (0.8 – 0.9)	0.1 (0.1 – 0.1)	0.8 (0.8 – 0.9)	0.8 (0.7 – 0.8)	0.0 (−0.2 – 0.1)	0.8 (0.8 – 0.8)	0.8 (0.8 – 0.9)	0.0 (−0.1 – 0.1)
Mobility right	0.0820	0.786	0.317	0.8 (0.8 – 0.8)	0.8 (0.8 – 0.9)	0.1 (0.1 – 0.1)	0.8 (0.8 – 0.9)	0.8 (0.8 – 0.9)	0.1 (−0.1 – 0.1)	0.8 (0.8 – 0.8)	0.8 (0.8 – 0.9)	0.0 (0.0 – 0.1)
Mobility full	0.0620	0.905	0.328	0.6 (0.6 – 0.7)	0.7 (0.7 – 0.8)	0.1 (0.0 – 0.1)	0.7 (0.7 – 0.9)	0.7 (0.7 – 0.8)	0.0 (−0.2 – 0.1)	0.7 (0.7 – 0.7)	0.7 (0.7 – 0.8)	−0.1 (−0.1 – 0.1)
