# Supplementary material for: Low-Intensity Virtual Reality Exercise for Caregivers of People with Mild Cognitive Impairment: A Pilot Study
Source: J Funct Morphol Kinesiol. 2025 Sep 16;10(3):353. doi: 10.3390/jfmk10030353 (PMC12452558; doi:10.3390/jfmk10030353)
Supplement: Supplementary file 1 [file jfmk-10-00353-s001.zip › Table S3.pdf]

Table S3. Differences between genders in clinical scales and instrumental measures. The table shows p-values from the Wilcoxon rank-sum test assessing gender differences (male, female) at T0, T1, and for delta scores for both clinical and instrumental outcomes. For each group and for each time point data are presented as median (1st – 3rd quartile). No correction for multiple comparisons was applied.

	Clinical scale	p-value T0	p-value T1	p-value Delta	Male T0	Female T0	Male T1	Female T1	Male Delta	Female Delta
	PSS	0.352	0.971	0.438	13.5 (8 – 17)	21 (15 – 24)	12 (10 – 18)	13 (9 – 18.5)	0 (−2 – 2)	−5 (−12 – 0.5)
STAI-Y	1	**0.048**	0.190	0.476	33.5 (32 – 36)	46.5 (38 – 55)	35 (26 – 43)	49 (36 – 50.5)	−1.5 (−5 – 2)	−5.5 (−10.5 – 4)
	2	**0.029**	0.190	0.448	33 (30 – 34)	45 (39 – 48)	32.5 (29 – 40)	43.5 (38 – 46.5)	−0.5 (−1 – 6)	−1.5 (−5.5 – 3)
	CBI	0.610	0.657	0.971	16.5 (10 – 23)	9 (1 – 24)	20 (12 – 28)	14 (6.5 – 23)	3 (−2 – 5)	−0.25 (−1 – 6)
	BDI-II	0.381	0.438	0.610	4.5 (2 – 11)	10.5 (5 – 17)	5.5 (1 – 10)	11 (4.5 – 18.5)	0 (−3 – 1)	0.5 (−1 – 2)
	SF-12	0.810	0.543	0.838	32.5 (30 – 34)	33.5 (26.5 – 34.5)	33 (32 – 33)	34 (26 – 36.5)	−0.5 (−2 – 3)	0.5 (−1.5 – 3)
COPE	Social Support	0.390	0.590	0.105	28 (24 – 33)	20 (16 – 29.5)	23.5 (20 – 26)	17.5 (14.5 – 29.5)	−4 (−8 – −3)	−0.5 (−3 – 1.5)
	Avoidance Strategies	0.810	0.314	0.886	20.5 (18 – 29)	20 (18.5 – 21.5)	20 (17 – 25)	18.5 (17 – 19.5)	−2 (−4 – −1)	−3 (−3 – −0.5)
	Positive Attitude	0.190	0.238	0.876	32.5 (31 – 39)	29 (21.5 – 32)	31 (30 – 38)	28 (21 – 31)	−0.5 (−2 – 3)	0.5 (−3.5 – 2)
	Orientation Problem	0.276	0.914	0.971	35.5 (34 – 36)	32.5 (21.5 – 35)	34.5 (29 – 38)	31.5 (21 – 37)	0.5 (−7 – 4)	−0.5 (−1 – 2.5)
	Transcendent Orientation	0.210	1.000	0.743	23.5 (18 – 26)	27.5 (24 – 30)	22 (21 – 24)	25 (20 – 30.5)	−0.5 (−2 – 3)	−1 (−5.5 – 2)
IPAQ	Vigorous IPAQ	0.743	0.648	0.181	2220 (0 – 5760)	4560 (2160 – 6000)	3840 (960 – 4320)	4320 (1440 – 3600)	−480 (0 – 2160)	−2160 (−3120 – −720)
	Moderate-IPAQ	0.752	0.810	0.524	1320 (720 – 3360)	1200 (0 – 3360)	2040 (480 – 5040)	720 (600 – 2880)	540 (0 – 1440)	−600 (−2640 – 2760)

	Walking-IPAQ	0.286	0.076	0.200	148.5 (0 – 660)	1188 (594 – 2673)	1085.7 (594 – 4158)	247.5 (49.5 – 891)	495 (297 – 4158)	−1138.5 (−1980 – −346.5)
	IPAQ	0.819	0.476	0.114	4950 (2457 – 10764)	8388 (4194 – 10593)	7156.2 (3036 – 9198)	2902.5 (1567.5 – 7173)	555 (2378.4 – 4617)	−2626.5 (−7690.5 – 1644)
	SUS	0.733	0.733	1.000	76.3 (67.5 – 85)	82.5 (73.75 – 90)	76.3 (67.5 – 85)	82.5 (73.75 – 90)		
	VAS	0.162	0.248	0.610	0.6 (0.0 – 1.33)	1.75 (1.41 – 2.11)	0.25 (0.0 – 1.14)	0.63 (0.59 – 1.0)	−0.5 (−1.33 – 0.31)	−0.83 (−1.46 – −0.48)
	Execution difficulty	0.143	1.000	0.381	1.0 (0.0 – 1.0)	0.0 (0.0 – 0.0)	0.0 (0.0 – 0.0)	0.0 (0.0 – 0.0)	0.5 (−1.0 – 0.0)	0.0 (0.0 – 0.5)
	Muscular difficulty	1.000	1.000	1.000	0.0 (0.0 – 1.0)	0.0 (0.0 – 0.5)	0.0 (0.0 – 1.0)	0.0 (0.0 – 0.5)	0.0 (−1.0 – 1.0)	0.0 (−0.5 – 0.5)
	Balance difficulty	0.524	0.810	0.819	1.5 (1.0 – 2.0)	1.0 (0.5 – 2.0)	0.0 (0.0 – 1.0)	1.0 (0.0 – 2.0)	−1.0 (−2.0 – −1.0)	0.0 (−2.0 – 1.5)
	**Instrumental data**									
	Mobility left	**0.019**	0.257	**0.038**	0.74 (0.7 – 0.77)	0.82 (0.8 – 0.9)	0.82 (0.8 – 0.88)	0.79 (0.74 – 0.81)	0.09 (0.05 – 0.11)	−0.04 (−0.16 – 0.02)
	Mobility right	**0.038**	0.610	0.114	0.8 (0.75 – 0.83)	0.84 (0.82 – 0.9)	0.86 (0.8 – 0.91)	0.82 (0.81 – 0.85)	0.05 (0.05 – 0.09)	−0.02 (−0.09 – 0.03)
	Mobility full	**0.038**	0.257	**0.038**	0.66 (0.62 – 0.7)	0.73 (0.71 – 0.82)	0.75 (0.7 – 0.79)	0.69 (0.67 – 0.71)	0.08 (0.02 – 0.11)	−0.05 (−0.15 – 0.0)
