# Supplementary figures and images for: Low-Intensity Virtual Reality Exercise for Caregivers of People with Mild Cognitive Impairment: A Pilot Study
Source: J Funct Morphol Kinesiol. 2025 Sep 16;10(3):353. doi: 10.3390/jfmk10030353 (PMC12452558; doi:10.3390/jfmk10030353)

**Figure S1.** Flowchart of the patients selection process.

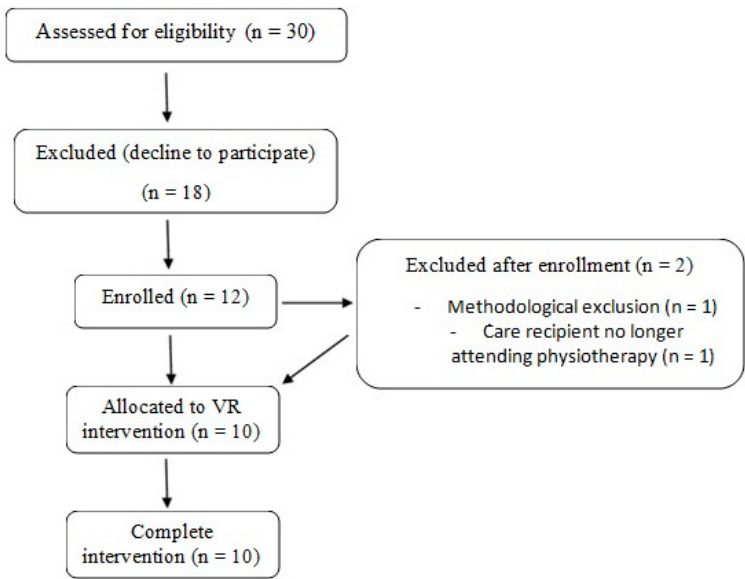

Supplement: Supplementary file 1 [file jfmk-10-00353-s001.zip › Figure S1.pdf]
